# Accuracy of intraoral real-time navigation versus static, CAD/CAM-manufactured pilot drilling guides in dental implant surgery: an in vitro study

**DOI:** 10.1186/s40729-022-00430-6

**Published:** 2022-10-06

**Authors:** Robert Stünkel, Alexander-Nicolai Zeller, Thomas Bohne, Florian Böhrnsen, Edris Wedi, David Raschke, Philipp Kauffmann

**Affiliations:** 1 Department of Maxillofacial Surgery, Georg August University, Göttingen, Germany; 2 Department of Maxillofacial Surgery, Hannover Medical School, Carl-Neuberg-Straße 1, 30625 Hannover, Germany; 3 Dental Practice, Northeim, Germany; 4 Department of Gastroenterology and Gastrointestinal Oncology, Interdisciplinary Endoscopy, University Medical Center, Georg August University, Göttingen, Germany

**Keywords:** Dental implants, Guided surgery, Stereotactic surgery, Referencing, Intraoral real-time navigation, Static templates, Implant accuracy

## Abstract

**Background:**

Nowadays, 3D planning and static for dynamic aids play an increasing role in oral rehabilitation of the masticatory apparatus with dental implants. The aim of this study is to compare the accuracy of implant placement using a 3D-printed drilling guide and an intraoral real-time dynamic navigation system.

**Methods:**

A total of 60 implants were placed on 12 partially edentulous lower jaw models. 30 were placed with pilot drilling guides, the other half with dynamic navigation (DENACAM^®^). In addition, implant placement in interdental gaps and free-end situations were investigated. Accuracy was assessed by cone-beam computed tomography (CBCT).

**Results:**

Both systems achieved clinically acceptable results, yet more accurate results regarding the offset of implant base and tip in several spatial dimensions were achieved using drilling guides (each *p* < 0.05). With regard to angulation, real-time navigation was more precise (*p* = 0.0016). Its inaccuracy was 3°; the template-guided systems was 4.6°. Median horizontal deviation was 0.52 mm at base and 0.75 mm at tip using DENACAM^®^. When using the pilot drill guide, horizontal deviation was 0.34 mm in the median and at the tip by 0.59 mm. Regarding angulation, it was found that the closer the drill hole was to the system's marker, the better navigation performed. The template did not show this trend (*p* = 0.0043; and *p* = 0.0022).

**Conclusion:**

Considering the limitations of an in vitro study, dynamic navigation can be used be a tool for reliable and accurate implantation. However, further clinical studies need to follow in order to provide an evidence-based recommendation for use in vivo.

**Supplementary Information:**

The online version contains supplementary material available at 10.1186/s40729-022-00430-6.

## Background

Today, the use of dental implants is an established procedure for rehabilitation of the masticatory apparatus after tooth loss [1–3]. Demands on functionality and esthetics have increased significantly overall [4, 5]. Current approaches are mostly based on a prosthetically driven treatment concept, the so-called backwards planning [6, 7]. Complex requirements for optimal implant positioning make profound preoperative and surgical-prosthetic planning necessary [8, 9]. The transfer of the planned implantological procedure into the operation room still poses the most challenging task and can be affected by numerous factors [10–13]. Devices for transferring position, angulation and alignment to other teeth and implants, can be drilling guides or real-time navigation systems [14–17]. These procedures can then be described as static and dynamic guidance. For static guidance, instruments without dynamic feedback, such computer aided designed (CAD) drilling guides are used. The term dynamic guidance is used for intraoperative real-time visualization to verify conformity of the conducted procedure with the preoperatively planned procedure [18]. Both can be summarized under the generic term of computer-aided surgery (CAS).

Drilling guides are usually produced by Computer Assisted Manufacturing (CAM) procedures such as 3D printing or milling [19, 20].

3D printers, such as the one used in this study by Stratasys^®^ (Stratasys GmbH, 77836 Rheinmünster, Germany), use so-called inkjet-based 3D printing techniques (also knowns as MultiJet or PolyJet) to build up the product layer by layer. These layers of light-sensitive polymer resins have a thickness of 0.02 mm and are sprayed on and directly light-polymerized. A roller system then thins them down to 0.016 mm, which is currently the smallest layer thickness of PolyJet systems [20]. Even in comparison with longer established stereolithography, PolyJet is highly precise [21]. Layer by layer, drilling guides are produced and can be subsequently refined and fitted with drilling sleeves.

Other than static guides, dynamic systems, such as navigation systems allow real-time monitoring of the bur position and drilling movements for implantology [17]. Dynamic navigation, compared to static drilling templates, allows visualization of the advancement in preparation of implant beds within the patients’ 3D data obtained from 3D-DICOM data. Deviations from the planned procedure thus become visible in real time. In comparison to drilling guides, adjustments can be made at any time in case of varying intraoperative circumstances [22]. For this, the operative situation, handpiece and navigation system are matched continuously to each other using optical tracking [23]. According to Block et al. [22], these systems can be differentiated into active and passive techniques. In active tracking, light is emitted by an apparatus attached to the handpiece, which is then captured by stereo cameras. For passive methods, tools such as recognition markers are used, which reflect light emitted by the system, back to the cameras [22].

For calibration of these systems, identification markers are necessary. They are usually radiopaque, so that they can be clearly identified in preoperative or intraoperative 3D X-ray data [24]. The computed tomography (CT) or cone-beam computed tomography (CBCT) is conducted with these markers in situ. Planning software can subsequently identify the marker in data sets. In most real-time navigation systems, these markers are usually partially or completely extraorally attached. Intraorally, markers can be adhesively fixed to teeth or dentures, or to osseous surfaces with mini screws [7, 15, 17]. Wang et al. [25] used a system that does not require a marker at all. It simultaneously records the patient's head and handpiece movements and computes corresponding spatial position. In other variants of navigation systems, separate recognition of the handpiece by the cameras installed in the room is mandatory. For this purpose, orientation elements can be mounted on the handpiece [26].

Prior to drilling, a registration process is used to detect the tip of the handpiece in its position in relation to the marker near the operating site and to match it to data [27, 28]. A simple calibration between a distinctive anatomical landmark, which is also clearly identifiable in the patient and in the CBCT, such as a fixed tooth, and the position in navigation device can verify accuracy before drilling begins [29]. Then the approximation to the planned position is demonstrated visually on the monitor. Crosshairs or bull's-eyes show targeted planning and, in relation to this, the distance of the drill tip in millimeters and the angle of the longitudinal axis. Furthermore, the reached drilling depth is digitally displayed [27]. Other than in static guide-based procedures, the precision of the procedure is continuously checked via the displayed data on the monitor and less via clinical situs inspection [30]. An ergonomically sensible posture and clear access to surgery areas with restricted mouth opening can thus be facilitated [30, 31]. Visualization of surgical situation can be achieved on conventional screens on the one hand site. On the other hand, this procedure could be combined with augmented reality with projectors or glasses [26]. In the literature, dynamically navigated surgery is currently attributed to come with by bulky tracking devices, inaccuracies in image registration procedures and with poor referencing of the patient to the navigation system [25].

Various factors impact on accuracy of real-time navigation systems. Artifacts in the CBCT caused by dentures, dental implants or non-removable body jewelry, as well as movements of the patient during image acquisition, can cause inaccuracies [32–34]. Furthermore, these initial inaccuracies increasingly correlate with the distance of the marker from the planned implant position, voxel size of the data set and implant length [35].

As it was not fully elucidated, whether dynamic or static methods of surgical guidance achieve more accurate results in implant positioning, this study was designed to compare the two methods. For this, the accuracies between the use of a surgical drilling guide supporting partial steps of implant drilling, and a real-time navigation system (DENACAM^®^ system, mininavident^®^) were evaluated. To account for the specific precision of implantations in different localizations and different relations to the recognition marker, it was chosen to simulate implantations in vitro in free-end situations and interdental gaps.

## Materials and methods

### Materials

**Fig. 1:**
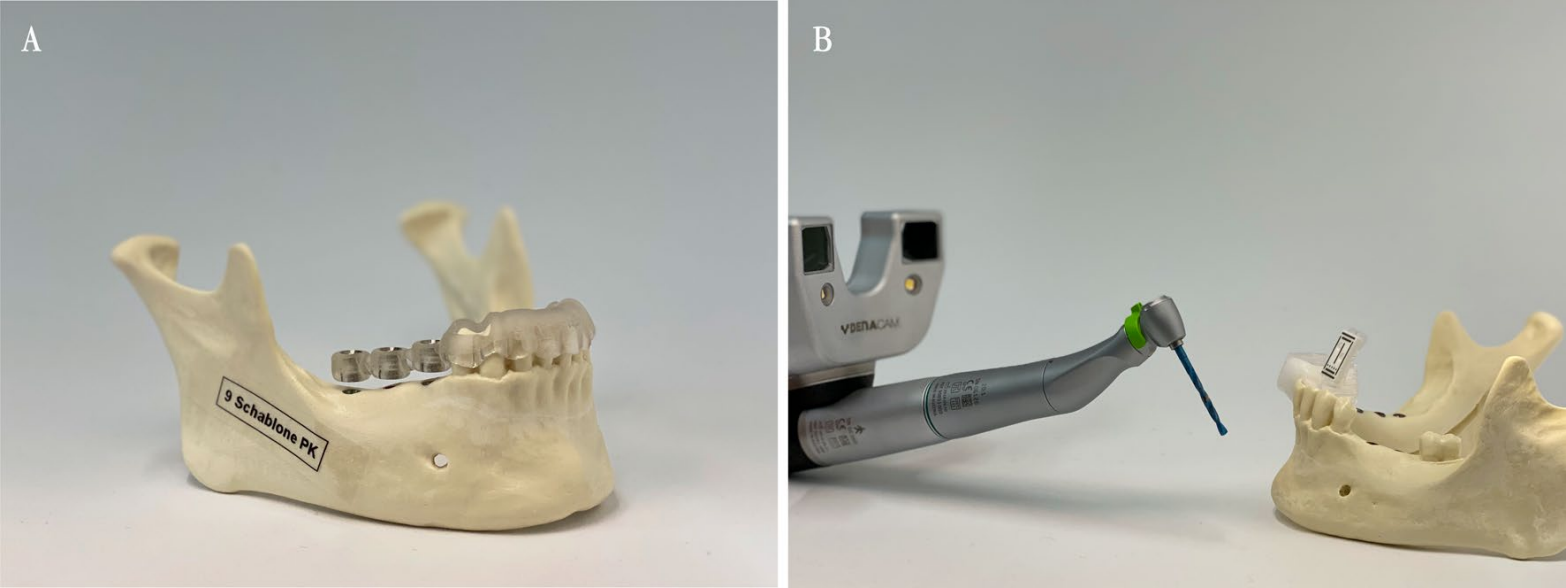
**A** mandible model used for the in vitro testing with pilot drilling guide in situ. **B** DENACAM system and mandible model with identification marker

**Fig. 2:**
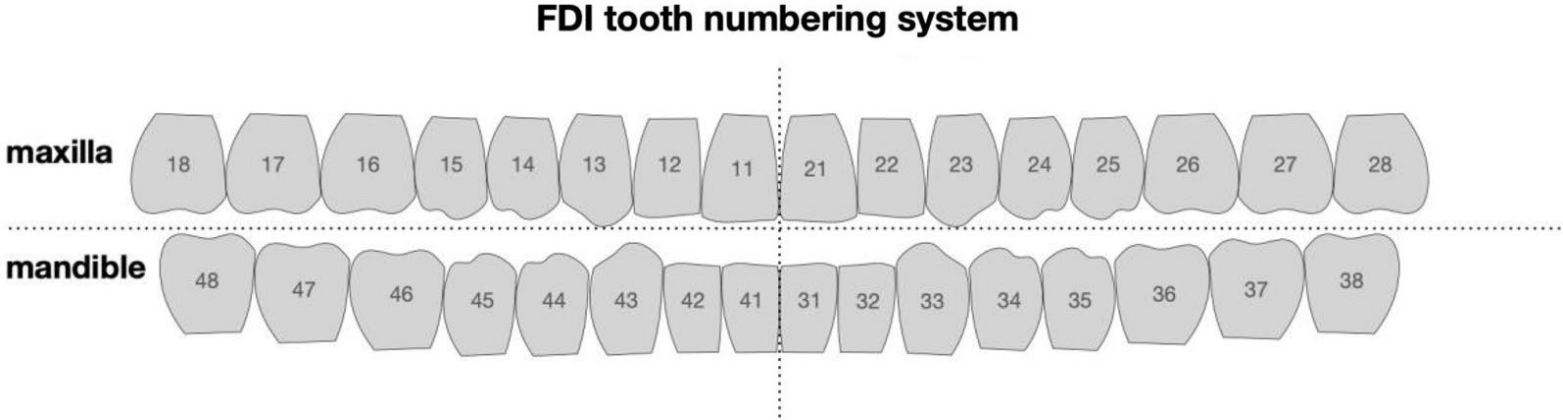
FDI tooth numbering system. The missing teeth in this study are 35, 36, 45, 46 and 47

To compare implantation procedures in vitro in free-end situations and interdental gaps, twelve anatomical plastic models (Mandibula partially edentulous Uni Göttingen Art. No. 1009", GOS^®^, Göttingen OP-Simulationssysteme, owner Dr. Thomas Bohne e.K., Northeim, Germany) were used (Fig. 1). The model is manufactured to simulate bone quality D1. Dentition of the model includes the remaining teeth 37, 34, 33, 32, 31, 41, 42, 43, 44 ("FDI notation", ISO 3950, Fig. 2).

A total of 60 implants were placed. Each five dental implants (Bone Level Implant ø 4.1 mm RC, 10 mm, Straumann^®^ AG, 4002 Basel, Switzerland) were placed per model in premolar and molar region. In all models the tooth positions 36, 35, 45, 46 and 47 were chosen.

### Digital planning

Models were scanned using CBCT with the PaX-Zenith 3D^®^ device from vatech^®^, Korea). The resolution was set to 0.3 mm voxels as the smallest displayable unit at voltage 120 kV, power 6 mA, exposure time 24,000 ms, width 160 mm and height 160 mm. Underlying digital planning for both, the static drilling guides and the navigation system, was performed with coDiagnostiX 9^®^ (Dental Wings GmbH, Chemnitz, Germany). The implants positions were chosen according to a future denture and were placed identically for all mandibulae. The drill sleeves were digitally aligned and added to the created template.

### Drilling guide-based group

Thirty implants in six mandibles were placed using drilling guides for the pilot drilling. For the CAD, guides that were digitally constructed based on the previously mentioned planning data were exported as .stl-files. These were then additively manufactured by PolyJet 3D printing with the CONNEX1 OBJET500^®^ (Stratasys GmbH, Rheinmünster, Germany) in an accuracy of 200 µm. The material used was biocompatible and has a tensile strength of 50–60 MPa as well as flexural strength of approximately 70 MPa. Universal sleeves (steco-system-technik^®^ GmbH & Co. KG, Hamburg, Germany) with an inner diameter of 2.35 mm and a length of 6 mm were integrated into the template. The template is rigidly anchored in the mouth via the remaining teeth (Fig. 1). First, the direction was set with the 2.2 mm diameter pilot drill #1. This fits seamlessly into the guide sleeve of the drilling template. This was followed by the pilot drill #2 (ø 2.8 mm) and the twist drill PRO^®^ (ø 3.5 mm) (Fig. 2). The planned length of 10 mm was always prepared at 800 rpm. In the system used, the sleeves were not changed with increasing diameters of the drills. It was assumed that the pilot hole is sufficient as a guide.

### Real-time navigation-based group

**Fig. 3:**
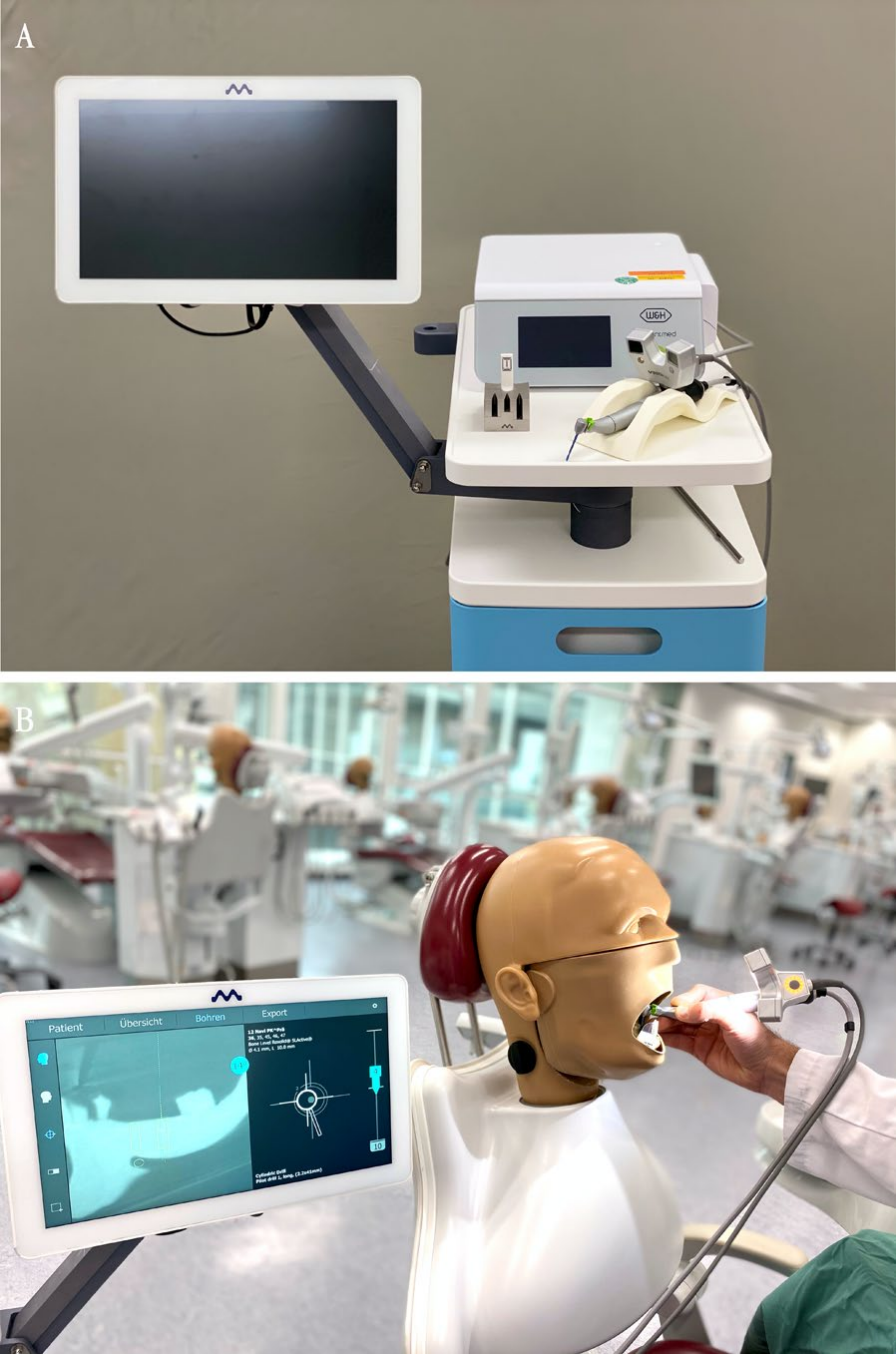
**A** DENACAM^®^ System, DENATOUCH® monitor, registration block, binoptical camera system on handpiece and DENACOMP^®^ computer unit. An additional foot pedal can be used to further control speed, water supply and drilling direction. **B** Right: DSEclinical 5197^®^ combination simulation unit (KaVo^®^) with elastic gingival mask. Left: the drilling process can be followed via DENATOUCH^®^ monitor in crosshair display

As for the drilling guide-based group, planning data were exported form the surgical planning software. For navigation, a commercially available system (DENACAM^®^ by mininavident^®^, Liestal, Germany) was used. In this system, the camera is mounted directly on the handpiece and the marker is fixed intraorally, contrary to common systems. This real-time navigation system uses a device for referencing, the DENATRAY^®^, which is fixed in the mouth with thermoplastic material. The DENAMARK^®^, a laser-engraved identification element, is attached to this fixture. The binoptical camera system (DENACAM^®^) is mounted onto the surgical handpiece and recognizes the position of the marker during surgery (Fig. 3). Thus, the position of the osteotomy can be followed in real time via the touch screen.

When using real-time navigation, each drill body is calibrated on the system directly before the respective osteotomy. Accordingly, the system is individually adjusted for each bur. By matching the data based on anatomical landmarks, such as a tooth, the correct registration can be checked before the drilling is performed.

### Implantation procedure and evaluation

**Fig. 4:**
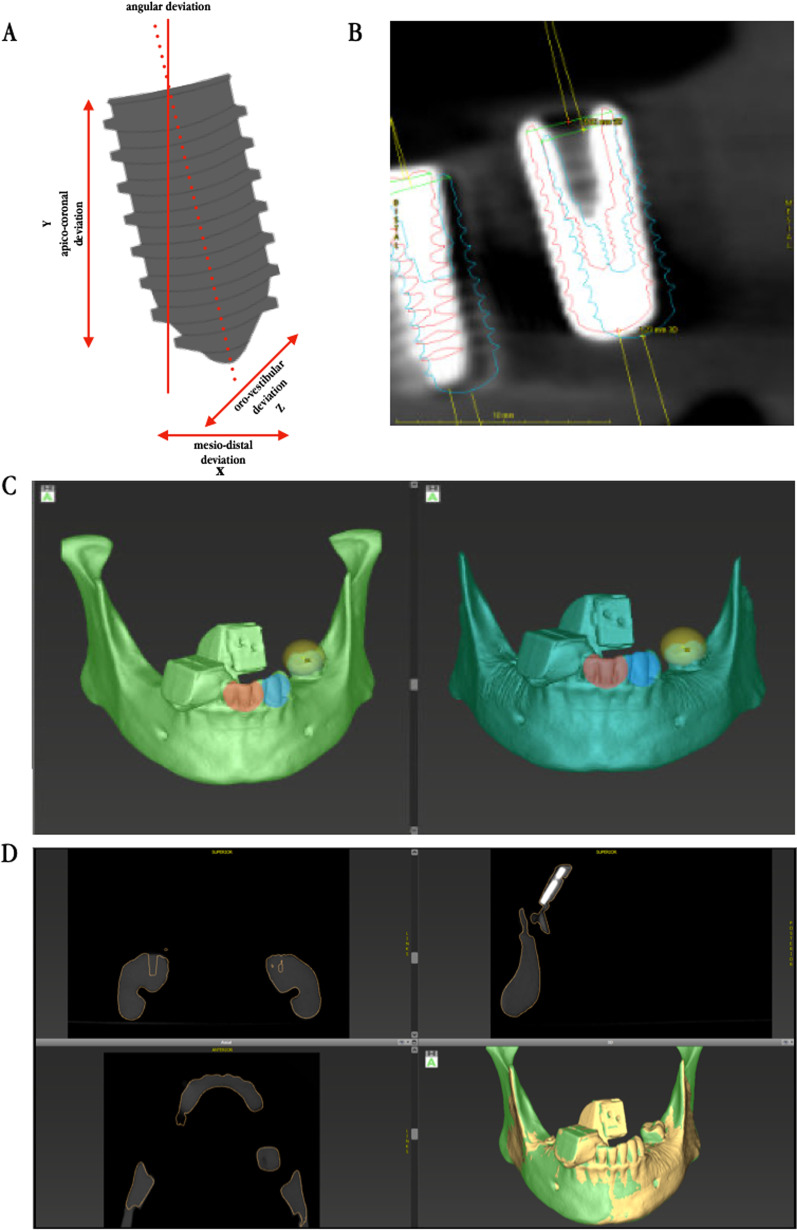
**A** Schematic illustration of spatial offsets from implant planning. Analysis shows deviations in drilling depth, oro-vestibular as well as mesio-distal direction and angulation. **B** Section of the Treatment Evaluation Tool in coDiagnostix^®^. Planning is shown in blue. Red shows the actual implant position. **C** Matching process of pre- and postoperative 3D data set by superimposition after selecting at least three congruent anatomical positions. **D** Graphical visualization of correlation of pre- and post-operative situation. Successful matching process can be verified in slices and in 3D reconstruction

For a realistic simulation, all implants were placed by two surgeons in dental simulation units (DSEclinical 5197^®^, KaVo Dental GmbH, Biberach, Germany). These units limit the mouth opening and access to the operating area by elastic face masks (Fig. 3). All implantations were completed according to the manufacturers drilling sequence wither with help of the navigation system or using the static drilling guide for the pilot drill. First, the time necessary to complete the procedures was recorded. After implantation, data for the evaluation of the achieved accuracy were acquired by using a postoperative CBCT. This data was re-imported into the “treatment evaluation” tool of coDiagnostix^®^. After fusion of the datasets, accuracy of the implantations was measured based on the displacements in *x-*, *y-* and *z-*axis at the implants tip and base as well as angulation. In detail, the spatial offset involved the deviation in apico-coronal, mesio-distal and oro-vestibular direction (Fig. 4).

Normal distribution of data was tested by Anderson–Darling, D’Agostino and Pearson, Shapiro–Wilk and Kolmogorov–Smirnov tests. For each, data were not normally distributed. Thus, statistical evaluation was conducted using the nonparametric Mann–Whitney *U* test in Prism 8^®^ Version 8.4.0 software (GraphPad Software, San Diego, CA, USA). To quantify deviation, measurements were adjusted for polarity sign. Results were non-parametrically ordered and were interpreted using median values (Additional file 1).

## Results

Main results of the deviation in *x*-, *y*- and *z*-axis, as well as angulation and required times, are summarized in Table 1. Median angular inaccuracy was significantly lower with the navigation system than with the pilot drill template (*p* = 0.0016). Navigated placed implants deviated median by 3° (median) from the planning, template-guided implants by 4.6°.

**Table 1:** Comparison of deviations when using real-time navigation and static pilot drilling template

n = 30/30	Navigation	Template	p-value	Significance
Angle	3°	4.6°	0.0016	*
Apical base	0.445 mm	0.275 mm	0.003	*
Apical tip	0.43 mm	0.23 mm	0.0006	*
Distal base	0.31 mm	0.285 mm	0.7662	
Distal tip	0.43 mm	0.48 mm	0.7888	
Vestibular base	0.42 mm	0.185 mm	0.0079	*
Vestibular tip	0.605 mm	0.34 mm	0.0341	*
3D offset base	0.8450	0.62 mm	0.0044	*
3D offset tip	0.9950	0.8250	0.2344	
Time	4.66 min (280 s)	3.175 min (190.5 s)	<0.0001	*

**Fig. 5:**
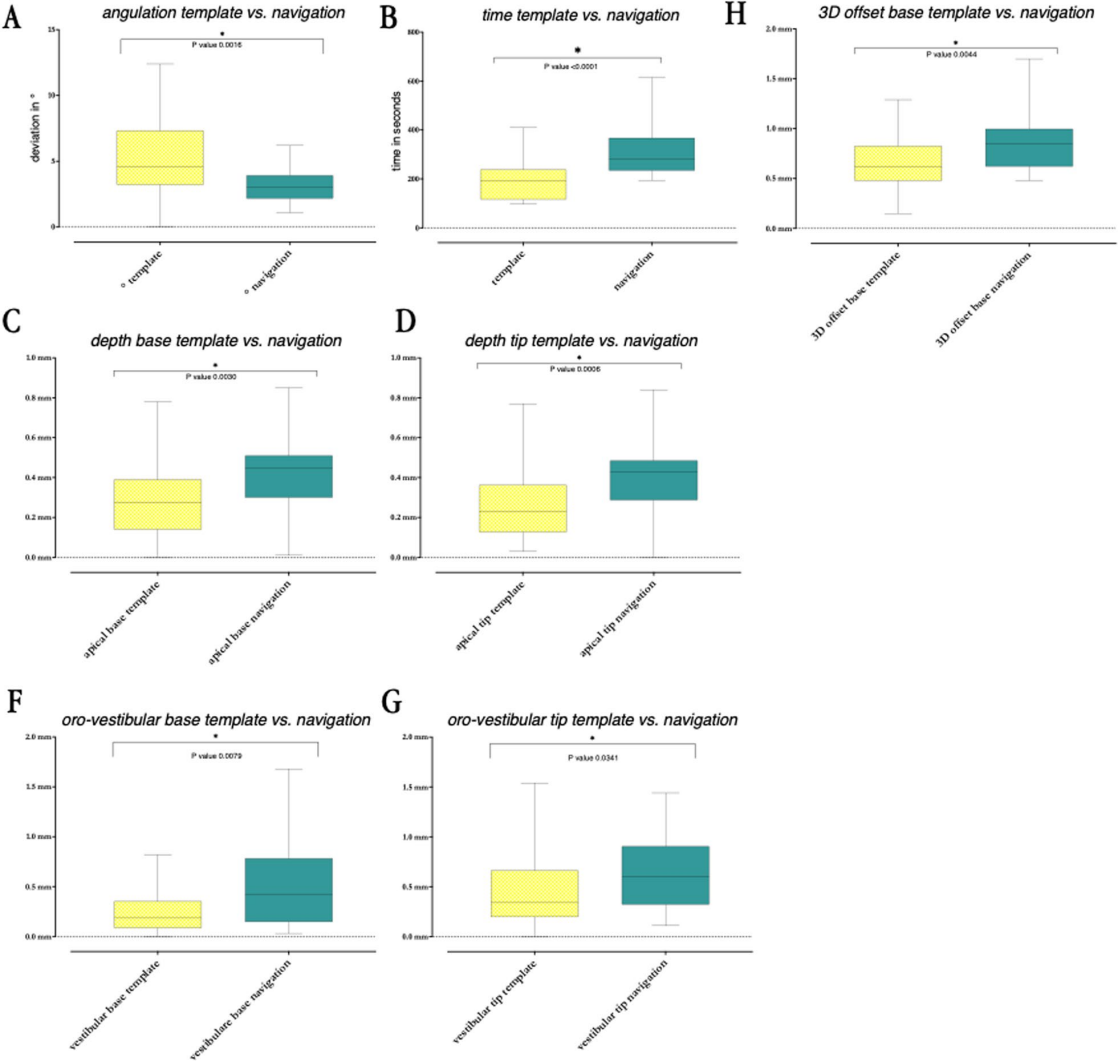
**A**–**G** Statistically significant (*) deviations in comparison between pilot drilling guide and intraoral real-time navigation with the DENACAM^®^ system. In each case, the distribution spectrum and the corresponding median value are shown. Each *n* = 60 (30/30)

At implant base and tip, deviations were significantly higher in vertical direction with the navigation system (*p* = 0.003, respectively, *p* = 0.0006). The base deviated 0.445 mm in median using navigation, and 0.275 mm with the template. The tip deviated 0.43 mm with real-time navigation and 0.23 mm with the pilot drill template. In oro-vestibular direction, the drill template-guided implants were significantly more accurate at base and tip (*p* = 0.0079, *p* = 0.0341). In mesio-distal direction there were no significant differences between the two systems at the implant base and tip (*p* = 0.7662, *p* = 0.7888). Median horizontal deviation was 0.52 mm at the implant base and 0.75 mm at the tip with the navigation system. The implant base, when using the pilot drill guide, deviated horizontally by 0.34 mm in the median and the tip by 0.59 mm. Placing implants by navigation required significantly more time than the guided pilot drilling (*p* < 0.0001). Median duration per implant was 4.66 min (280 s) for real-time navigation. With the guide, one implant required 3.18 min (190.5 s) (Fig. 5).

**Fig. 6:**
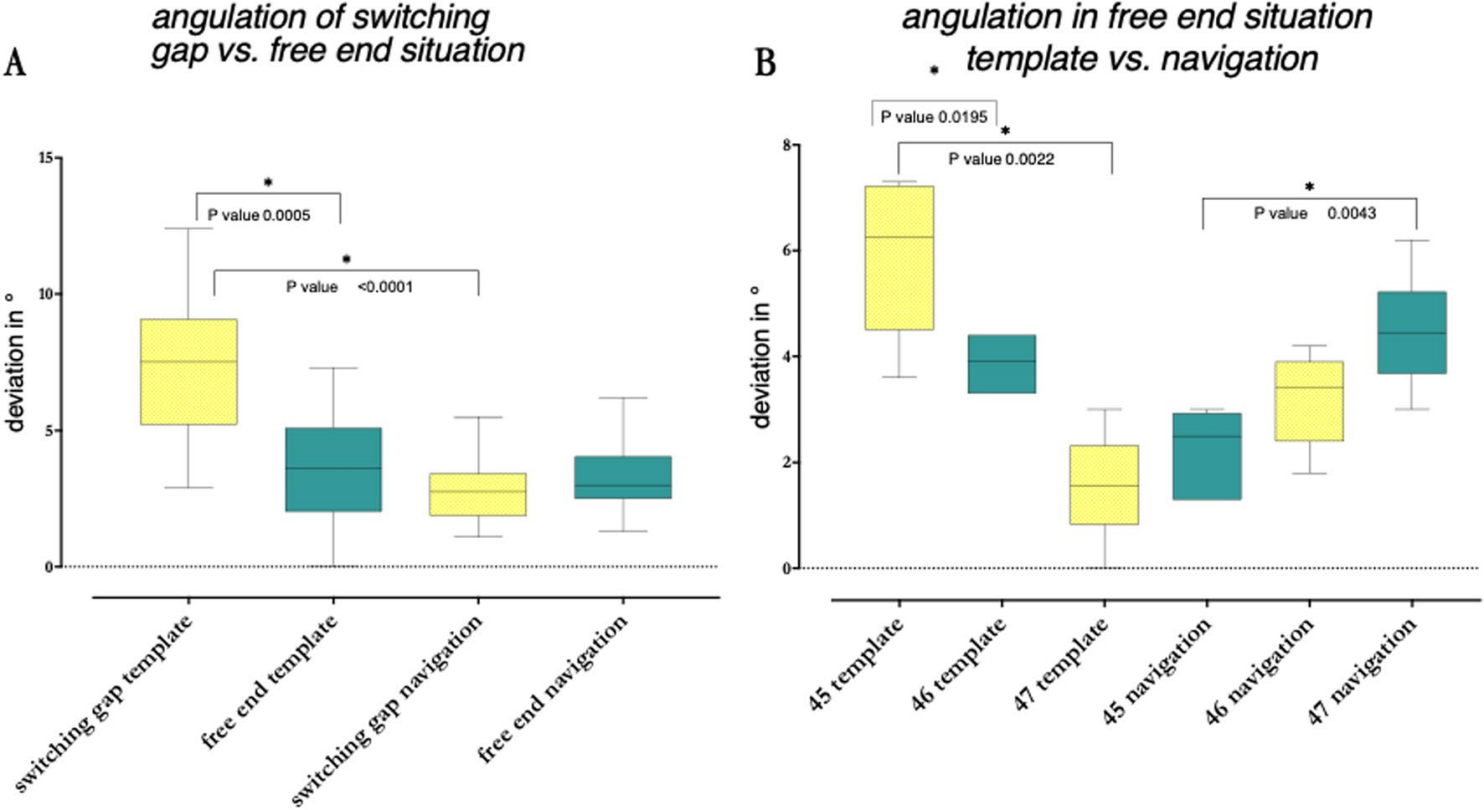
**A** Comparison of the angulation in interdental gap (positions 35 and 36) against free end (positions 45, 46 and 47). *n* = 60 (12/18/12/18). **B** Comparison of the angulation within the free end positions (45, 46 and 47) to show increasing inaccuracies with growing distance to the marker. In each case, the distribution spectrum and the corresponding median value are shown. Statistically significant values are shown with an asterisk (*). *n* = 36 (6/6/6/6/6/6)

In comparison, the spatial position of the implant base was significantly more accurate (*p* = 0.0044) when the implants were placed by static guidance. Median position deviated by 0.845 mm for the navigation system. Using the guide, median position differed by 0.62 mm. Spatial offset of the implant tip was not significantly different (*p* = 0.2344). The navigation group deviated 0.995 mm in median from planned position. The pilot drill guided group deviated by 0.825 mm. As distance from the marker to the implant increased, the inaccuracy in terms of the angle grew for the navigation system (*p* = 0.0043). In spatial offset, this observation did not exist (*p* = 0.6691, respectively, *p* = 0.5887). In the interspace gap (regions 35 and 37) it was observed that regarding angulation, implants were placed more accurately with real-time navigation than with the pilot-drill guide (*p* < 0.0001). At the free end (region 45, 46, 47), the guided procedure was significantly more accurate in terms of the spatial precision of implant base and tip (*p* = 0.0053, *p* = 0.0308) (Fig. 6).

## Discussion

The aim of this study was to compare the accuracy of a new, entirely intraoral, real-time navigation system for dental implantology with the clinically established method of static pilot drilling guides. In many institutions, pilot drilling guides are an established method, as they are inexpensive in production on the one hand. On the other hand, this method leaves the surgeon with the possibility to react to unexpected intraoperative situations due to the not fully guided drilling sequence. Although a possibly higher accuracy with a fully guided drill guide, this method has its justification as it is time-saving and economic. Due to these advantages, pilot drilling templates are used very frequently and were therefore used as a comparative methodology in our case. The study was performed in vitro on simulation units with elastic face masks. In a review by Gargallo-Albiol et al. [36], it was found that most of the work on dental real-time navigation described so far, is based on in vitro studies. According to Tahmaseb et al. [37], overall deviations observed in clinical in vivo studies are significantly higher than in in vitro experiments. In vivo, anatomical limitations such as tongue, cheek and mouth opening are factors that should be considered in an evaluation of the practicability of real-time navigation systems. Other factors such as bleeding, disturbing amounts of saliva and differing bone quality are also not included in model studies [38]. For comparison of two methods in vitro, it can be assumed, that the inaccuracies arising from these factors are similar for both methods. Further conflicts for precise navigated implantation can arise from prolonged delays between planning and surgery. In a clinical study by Block et al. [39], the authors reverted to free-hand technique intraoperatively after the recognition markers no longer fitted correctly due to new dental restorations or an in other ways changed clinical situations. Other authors used similar models or additional gingival masks in order to simulate a situation, that is as close to reality as possible but at the same time remaining reproducible [18, 28]. For the DENACAM^®^ system, so far only very few studies regarding its accuracy existed [40, 41]. They suggested similar accuracies to static guided pilot drilling, yet did not discriminate between free end position and interdental gaps. In order to objectively carry out a detailed comparison of both methods with each others, the before mentioned method was used.

Implant position is crucial for longevity of implant-supported dental restorations [28]. Precise planning is particularly useful for difficult anatomies or special indications [3]. Especially for cases of severe atrophy, extensive augmentative procedures may be necessary prior to implantation. To reduce costs, the morbidity of the procedure and the time from implantation to oral rehabilitation, there are approaches to avoid bone augmentations. In the maxilla, dental implants can be placed at an angle, so-called off axis, to prevent perforation of the Schneider's membrane of the sinus. This method is technically more complicated, but known to still achieve sufficient osseointegration [42]. Similar to this, in the mandible, augmentative procedures often can be avoided, if high precision of the surgical procedure can be guaranteed for. The literature describes maintaining safety distances around crucial anatomical structures to be useful [14, 43–48]. Inaccuracies, such as those specified in our case of the X-ray unit, the PolyJet system as well as the evaluation software, which is even subject to individual assessment, add up additionally to the intraoperative accuracy discrepancies. Using static guidance systems, safety distances of 2–4 mm to important anatomical structures have been required by some authors. For critical situations as those described before, narrow spaces as well as converging root anatomies of adjacent teeth, using a real-time navigation has been postulated to be advantageous [18]. Furthermore, navigation system has advantages in cases where space is limited, such as in the mandibular anterior region, where static guides might be too large for using them [30]. Considering the radius surrounding planned implant positions, also described for the navigation system, a safety distance to relevant structures of 1–2 mm has been described as advisable [18, 22]. Our figures showed a median deviation of 3° in angulation when using the navigation system. The implant base deviated 0.445 mm vertically and 0.43 mm at tip. Horizontal deviation was 0.52 mm at the base and 0.75 mm at apex. On basis of the data collected in this study and taking into account other potential inaccuracies, a safety margin of at least one millimeter might therefore be reasonable in order not to damage crucial structures.

Implants in this study were placed significantly less accurate in the most distal position 47 regarding angulation than implants placed using drilling guides (*p* = 0.0047). Regarding 3D offset of base and tip, the navigation system was also significantly less accurate in the free-end situation (*p* (base) = 0.0053, *p* (tip) = 0.0308). Results from the literature are in concordance with our observations [35]. According to manufacturers' instructions, the literature discussed and our results, it can be assumed that implants placed in positions far from the marker can become significantly less accurate, particularly in procedures with the placement of a multitude of implants [49]. In addition to a safety distance, the prosthetic goal should be carefully reviewed for implant positions far distant to the marker. Especially if an immediate restoration is desired, a more generous tolerance should be planned for distal implants than for implants closer to the marker.

Compared to free-hand implant placement, real-time navigation is known to be superior in terms of accuracy [14, 36, 39, 50–53]. In this experiment, results were obtained that are similar to those described in the literature [29, 51, 52, 54]. Casap et al. [55] examined an image-guided implantology system for its accuracy by measuring point analysis and determined a mean spatial displacement of 0.35 mm. For deviations over 0.75 mm, the probability was 0.003 and for those over one millimeter 0.0001. Chiu et al. [54] performed 80 real-time navigated implantations in artificial jaws. They found a mean horizontal deviation of the implant base of 0.43 mm and an angulation inaccuracy of 4°. Two-thirds of their drillings had maximum deviations in depth of 1 mm. One-third had been drilled to a maximum depth of 1.04 mm and had perforated the model’s mandibular canal. Kramer et al. [52] compared 40 implants each with navigated and conventional technique. In horizontal position maximum deviations of 0.6 mm were shown with the navigated approach. Angulation varied by a maximum of 8°. At planned depth, navigated implants were inaccurate by a maximum of 0.4 mm. A comparison of two navigation systems showed larger deviations at the implant tip compared to the base (*p* (tip) = 0.0023, *p* (base) = 0.0001). At the base, both systems deviated 0.37 mm to 0.65 mm, at the tip 0.47 mm to 0.68 mm [51]. Depth deviation between these systems varied between 0.32 mm and 0.61 mm. In a study by Stefanelli et al. [29] 231 implants placed under the use of navigation were evaluated in patients between 2015 and 2017. There were deviations of 0.4 mm for the base and 1.0 mm for the implant tip. Mean angular deviation was 2.26°. This is overall consistent with the data collected in this study. Interestingly, most angular deviations using navigated procedures are similar to those found in the presented data. Wu et al. found similar values in an in vivo study, retrospectively comparing implant placing precision with static and dynamic guidance in 2020 [56]. Within their group, navigation performed superior to static guidance with respect to angulation in the molar region. Unfortunately, there is no explicit discrimination between interdental gap and free end positions.

Even though, static and dynamic guidance are known to be superior over free-hand placing of implants, they also come with the necessity of a CT or CBCT [53]. It has been pointed out by some authors, that these procedures are not always necessary and that “the temptation to utilize 3-D imaging in every implant placement” should be resisted [53].

Undisputedly, this in vitro study has some limitations. Apart from the fact, that conclusions from in vitro generated data have to be carefully reviewed before using them for clinical decisions, also the study design comes with some limitations. The detected aberrances have been close to the resolution of the CBCT used, thus posing a possible source of error. More precise evaluation though, for example by micro-CT, seems disproportionate, as the hereby increased resolution would only be able to show aberrances of a not clinically significant extent.

Due to its compact design, DENACAM^®^ is completely new compared to conventional real-time navigation systems [57]. In other systems, the camera unit is mounted on the ceiling, extends from swivel arms or is mounted on tripods in the operating room [16, 18, 58–60]. The more portable design with intraoral recognition marker may allow application of real-time navigation with a manageable amount of equipment in general practice.

## Conclusion

In conclusion, both methods, the intraoral real-time navigation method with intraoral markers and the pilot drill guides are a reliable tool for transferring planned implant positions into the patient, at least in vitro. Both came with clinically acceptable inaccuracies regarding angulation and positioning of the dental implants. With regard to the cost–benefit ratio, high acquisition costs for navigation systems are in contrast to comparatively low production costs and simple manufacturing of conventional template systems. Furthermore, the manufacturing of guides can be delegated to dental laboratories, the set-up of the navigation system is always up to the clinical staff.

It has to be considered though, that navigation systems can be useful for surgery in areas where it is difficult to place guides. Furthermore, its use may be advantageous especially for young dentists, as it can objectively simulate surgical results.

The presented inaccuracies justify the use of both systems. Further clinical studies should therefore be performed to establish recommendations regarding situations for the clinical use of real-time navigation with intraoral markers.

## Supplementary Information

**Additional file 1: Fig. S1.** A) Schematic illustration of spatial offsets from implant planning. Analysis shows deviations in drilling depth, oro-vestibular as well as mesio-distal direction and angulation. B) Section of the Treatment Evaluation Tool in coDiagnostix^®^. Planning is shown in blue. Red shows the actual implant position. C) Mandible model used for the in vitro testing with pilot drilling guide in situ. D) DENACAM system used for the in vitro testing. E) Major findings in absolute numbers.

## Data Availability

The datasets used and/or analyzed during the current study are available from the corresponding author on reasonable request.

## Abbreviations

3D: Three-dimensional
CAD: Computer-aided design
CAM: Computer-aided manufacturing
CAS: Computer-aided surgery
CBCT: Cone-beam computed tomography
CT: Computed tomography
DICOM: Digital imaging and communications in medicine
FDI: Fédération Dentaire Internationale
