# Supplementary material for: Accuracy of intraoral real-time navigation versus static, CAD/CAM-manufactured pilot drilling guides in dental implant surgery: an in vitro study
Source: Int J Implant Dent. 2022 Oct 6;8:41. doi: 10.1186/s40729-022-00430-6 (PMC9535055; doi:10.1186/s40729-022-00430-6)
Supplement: Supplementary file 1 — Additional file 1: Fig. S1. A) Schematic illustration of spatial offsets from implant planning. Analysis shows deviations in drilling depth, oro-vestibular as well as mesio-distal direction and angulation. B) Section of the Treatment Evaluation Tool in coDiagnostix®. Planning is shown in blue. Red shows the actual implant position. C) Mandible model used for the in vitro testing with pilot drilling guide in situ. D) DENACAM system used for the in vitro testing. E) Major findings in absolute numbers. [file 40729_2022_430_MOESM1_ESM.pdf]

A B C D

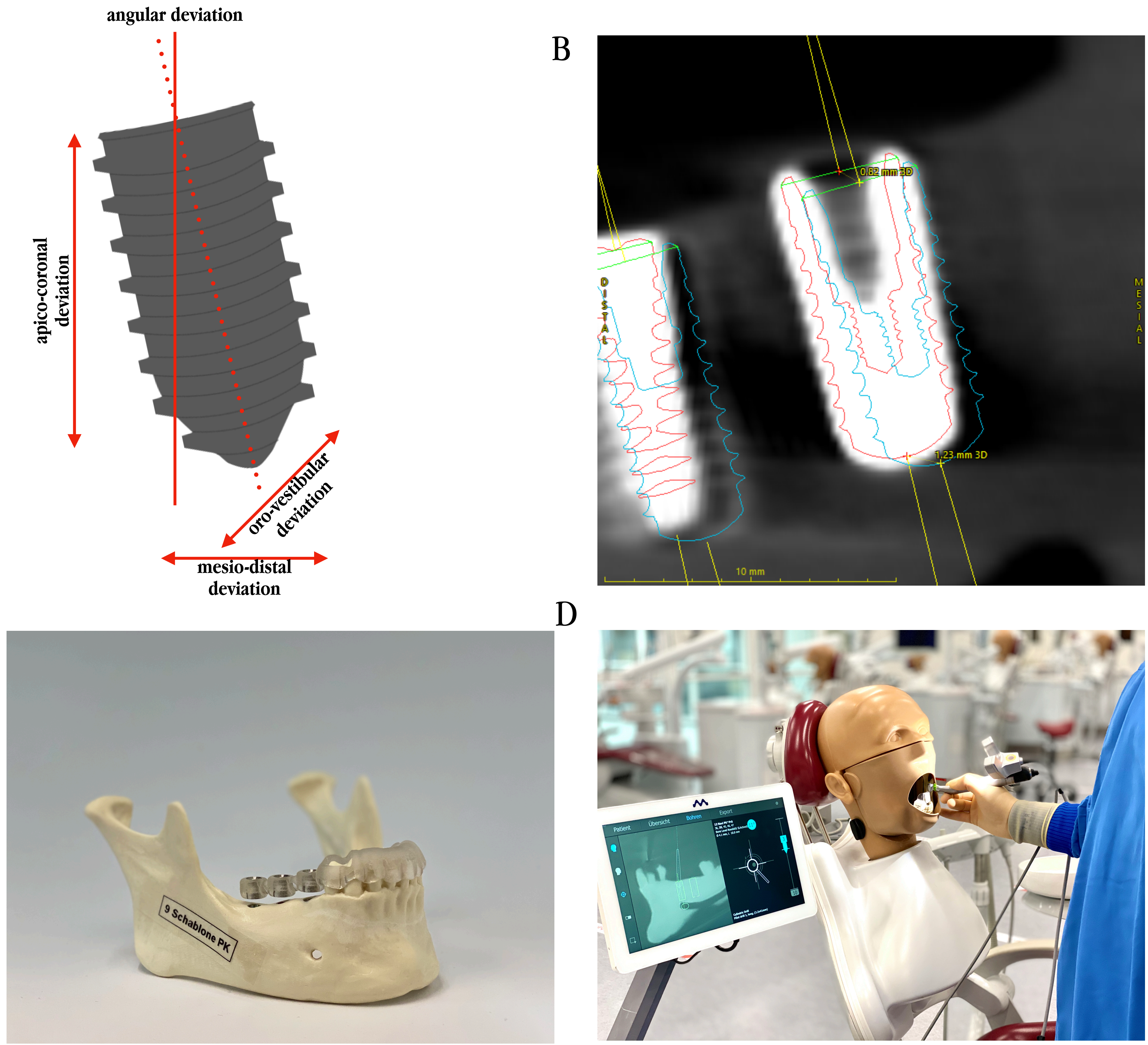

E

	drilling template	navigation
angular deviation	4.6°	3°
median horizontal deviation base	0.34 mm	0.52 mm
median horizontal deviation tip	0.59 mm	0.75 mm
median vertical deviation base	0.275 mm	0.445 mm
median vertical deviation tip	0.23 mm	0.43 mm
median duration per implant	3.18 min	4.66 min

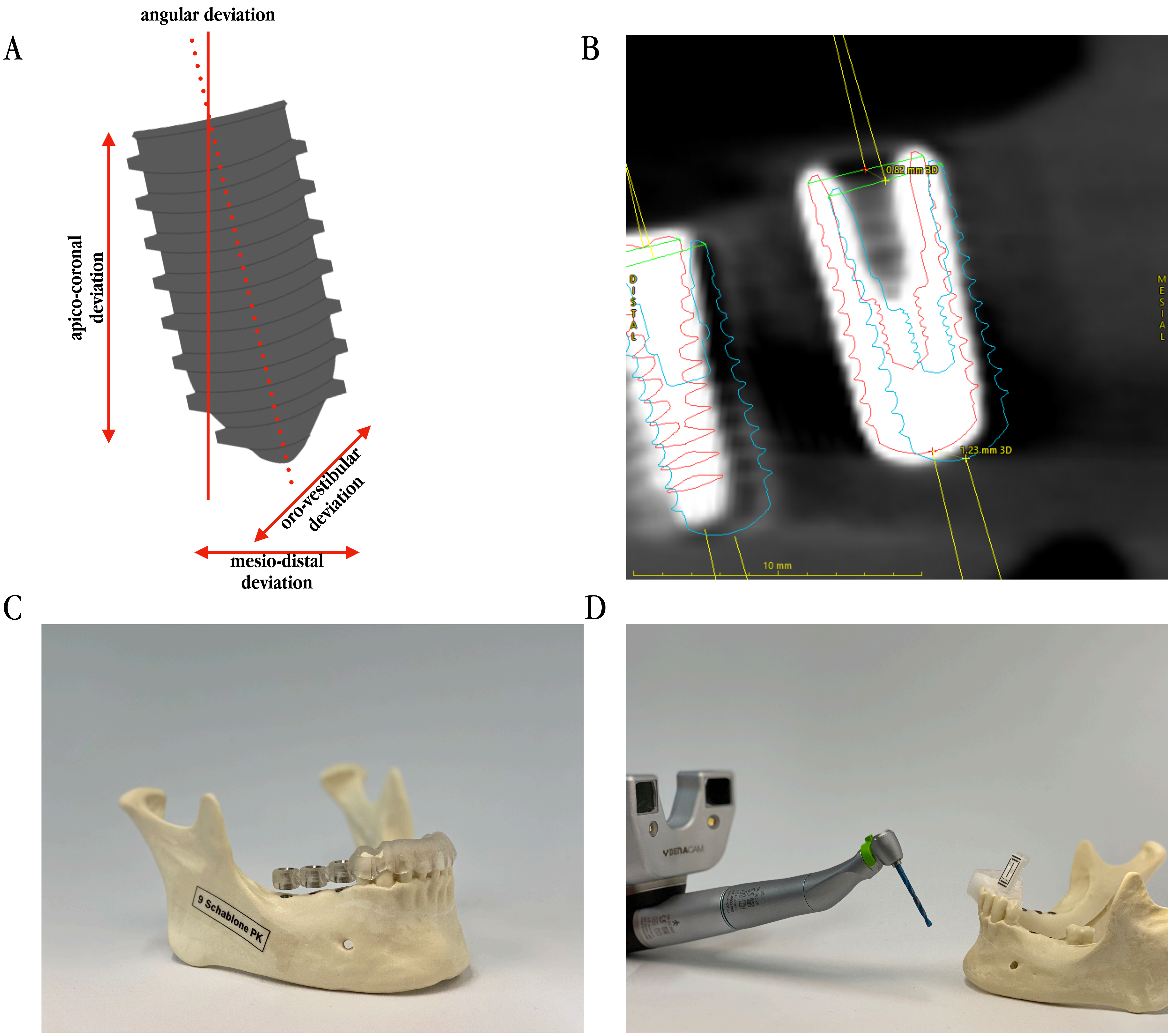

E

	drilling template	navigation
angular deviation	4.6°	3°
median horizontal deviation base	0.34 mm	0.52 mm
median horizontal deviation tip	0.59 mm	0.75 mm
median vertical deviation base	0.275 mm	0.445 mm
median vertical deviation tip	0.23 mm	0.43 mm
median duration per implant	3.18 min	4.66 min
